# Inhibition of epidermal growth factor receptor attenuates LPS-induced inflammation and acute lung injury in rats

**DOI:** 10.18632/oncotarget.15790

**Published:** 2017-03-01

**Authors:** Xiaoou Shan, Yali Zhang, Hongjin Chen, Lili Dong, Beibei Wu, Tingting Xu, Jie Hu, Zhiguo Liu, Wei Wang, Liqin Wu, Zhiguo Feng, Guang Liang

**Affiliations:** ^1^ Chemical Biology Research Center at School of Pharmaceutical Sciences, Wenzhou Medical University, Wenzhou, Zhejiang, China; ^2^ The Second Affiliated Hospital and Yuying Children's Hospital of Wenzhou Medical University, Zhejiang, China; ^3^ Children's Hospital of Zhengzhou, Zhengzhou, Henan, China; ^4^ School of Pharmacy, Qingdao University, Qingdao, Shandong, China

**Keywords:** acute lung injury, epidermal growth factor receptor, inflammation, lipopolysaccharide, mouse peritoneal macrophages

## Abstract

Acute lung injury (ALI) and its severe form acute respiratory distress syndrome remain the leading cause of morbidity and mortality in intensive care units. Inhibition of epidermal growth factor receptor (EGFR) has been found to be able to reduce inflammatory response. However, it is still unclear whether EGFR inhibition can prevent ALI. This study aimed to validate the EGFR's role in ALI and investigated the effects of EGFR inhibition on lipopolysaccharides (LPS)-induced ALI in rats. *In vitro*, both pharmacological inhibitors (AG1478 and 451) and si-RNA silencing of EGFR significantly inhibited LPS-induced EGFR signaling activation and inflammatory response in human lung epithelial cells or macrophages. Mechanistically, LPS induced EGFR activation via TLR4 and c-Src signaling. *In vivo*, rat model with ALI induced by intratracheal instillation of LPS was treated by oral administration of AG1478 and 451. It was observed that AG1478 and 451 blocked the activation of EGFR signaling in lung tissue and reduced the LPS-induced infiltration of inflammatory cells, inflammatory gene expression, and lung injuries. This study demonstrates that TLR4/c-Src-dependent EGFR signaling plays an important role in LPS-induced ALI, and that EGFR may be a potential target in treating ALI.

## INTRODUCTION

Acute lung injury (ALI), known as acute respiratory distress syndrome (ARDS), is a severe, life-threatening medical condition characterized by acute and widespread inflammation in the lungs [1]. While ALI may be triggered by trauma or infection, it is usually the result of sepsis. Inflammation, such as that caused by sepsis, produces endothelial dysfunction, fluid leakage from the capillaries and impaired drainage of fluid from the lungs [2]. Even with more advanced and improved supportive care and better understanding about the pathogenesis and development of ALI/ARDS, the syndrome has a high mortality between 30 and 40% [3, 4]. None of the pharmacological treatments evaluated in Phase II and Phase III for ALI/ARDS has been proven to be effective [5]. Thus, there is an urgent need to research new therapeutic targets and agents for the treatment of ALI.

EGFR, a tyrosine kinase receptor, can be activated by direct ligand binding or transactivated by a variety of infectious and noninfectious endogenous stimuli. EGFR activation leads to the activation of a number of downstream signaling cascades, such as extracellular regulated kinase (ERK) and phosphoinositide-3 kinase (PI3K)/Akt, which are important in the regulation of cell proliferation, survival, differentiation, migration, and matrix homeostasis in normal and pathological states such as cancer [6]. As a result, EGFR has been increasingly targeted as an important therapeutic option. In the past two decades, more than 7 EGFR inhibitors have been clinically used for cancer treatment [7–9]. Additionally, it was found that EGFR plays an important role in non-malignant disorders and EGFR inhibitors attenuate the metabolic diseases both *in vivo* and *in vitro* studies through anti-inflammatory and anti-oxidative actions [10–13].

While EGFR inhibition has been found to be able to reduce inflammatory response induced by various stimuli, limited data are available for the role of EGFR in the pathogenesis of ALI. It was found that after naphthalene-induced airway injury, EGFR and EGF expression levels are increased in the epithelial cells of distal bronchioles [14]. A study showed that EGFR regulated mechanical ventilation-induced lung injury, and EGFR inhibitor AG1478 markedly attenuated both lung alveolar and vascular permeability, accompanied by diminished levels of lung inflammatory responses [15]. This work indicated a detrimental role of EGFR in the pathogenesis of ALI. However, surprisingly an independent study from Chika Harada's group showed that treatment with the EGFR inhibitor gefitinib after naphthalene prolonged neutrophil sequestration and worsened ALI in mice, indicating a contributing role of EGFR activation in ALI [16]. Thus, these two opposite results suggest that EGFR's role in the development of ALI is complicated and requires further deeper demonstration.

In this study, we investigated the effects of EGFR inhibition on lipopolysaccharides (LPS)-induced ALI in rats. In addition, we evaluated the anti-inflammatory effects of EGFR inhibition or silence *in vitro*, as well as the mechanism of LPS-induced activation of EGFR.

## RESULTS

### EGFR inhibitors AG1478 and 451 blocked LPS-induced EGFR signaling activation and inflammatory cytokine production in macrophages

**Figure 1:**
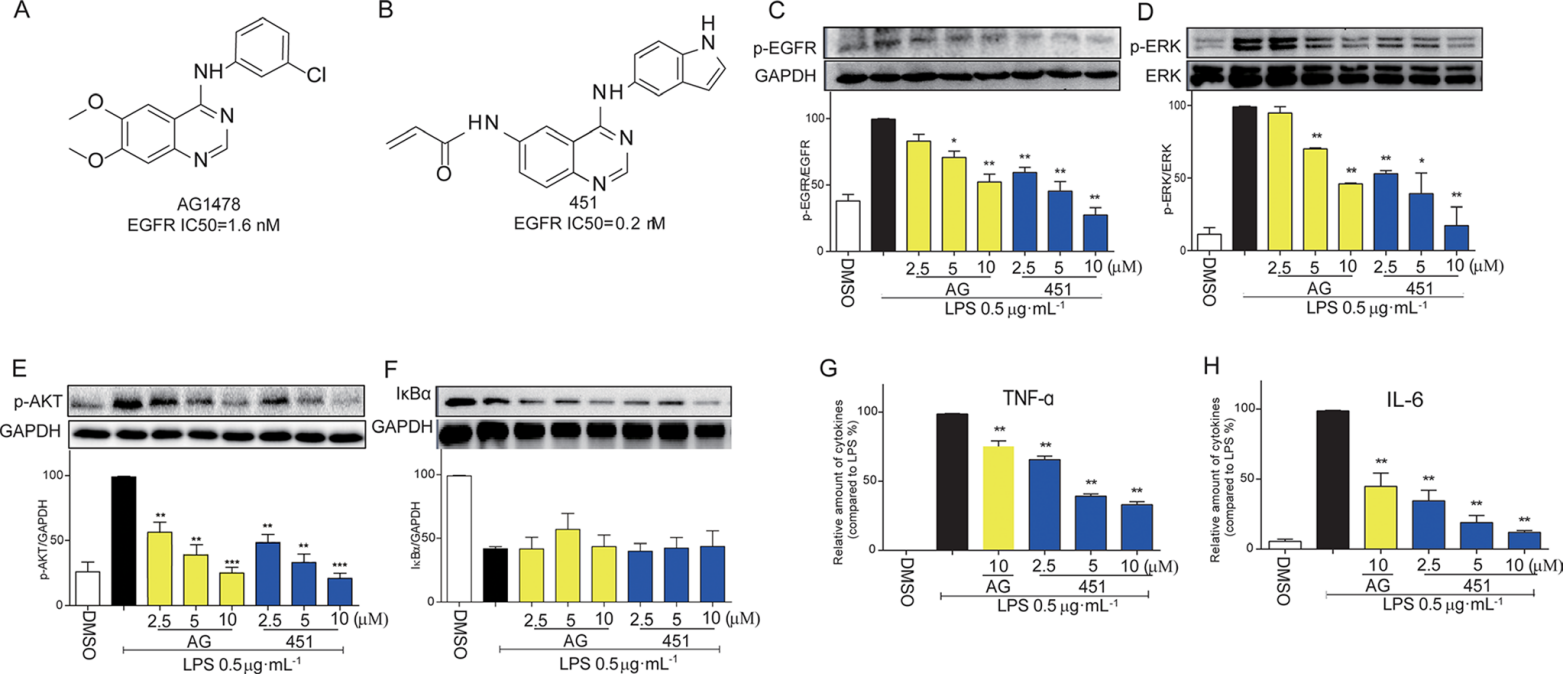
EGFR inhibitors inhibited LPS-induced EGFR signaling pathway and inflammatory production in macrophages (**A**) The chemical structure of EGFR inhibitor AG1478. (**B**) The chemical structure of EGFR inhibitor 451. (**C**–**F**) MPMs were pre-treated with AG1478 or 451 at various doses (2.5, 5, 10 μM) or vehicle (DMSO) for 30 min prior to stimulation with LPS (0.5 μg·mL^−1^) for 30 min. The total protein was collected and the levels of p-EGFR (C), p-ERK (D), p-AKT (E), and IκBα (F) were detected by Western Blot assay. (**G**–**H**) MPMs were pre-treated with AG1478 at 10 μM or 451 at various doses (2.5, 5, 10 μM) or vehicle (DMSO) for 30 min prior to stimulation with LPS (0.5 μg·mL^−1^) for 24 h. ELISA assay was used to detect the production of inflammatory cytokine TNF-α (G) and IL-6 (H) in medium. Bars represent the mean ± SEM of more than three independent experiments performed in duplicate, and asterisks indicate significant inhibition (*p < 0.05, and **p < 0.01, vs. LPS group).

Two known EGFR small-molecule inhibitors, AG1478 (Figure 1A) and 451 (Figure 1B), [13, 17] were used to block EGFR signaling activation in mouse peritoneal macrophages (MPMs). Firstly, we tested the anti-inflammatory effects of EGFR inhibitors in LPS-stimulated MPMs. Upon LPS stimulation, macrophages developed the activation of EGFR signaling pathway, such as EGFR phosphorylation (Figure 1C), ERK phosphorylation (Figure 1D), and AKT phosphorylation (Figure 1E). Pretreatment with AG1478 or 451 inhibited the activation of EGFR, AKT and ERK in a dose-dependent manner (Figure 1C–1E). As observed in molecular level, 451 showed a stronger inhibition on EGFR signaling activation. LPS stimulation also significantly induced NF-κB activation, a classic transcriptional factor regulated by LPS-TLR4 pro-inflammatory signaling, as evidenced by the degeneration of IκBα (Figure 1F). However, AG1478 and 451 failed to reverse LPS-induced IκBα degeneration, indicating that LPS-induced EGFR signaling pathway was irrelated to NF-κB. We then examined the anti-inflammatory effects of EGFR inhibitors. The stronger one, 451, was used dose-dependently, while AG1478 only at 10 μM. As shown in Figure 1G and 1H, pretreatment with 451 significantly inhibited LPS-induced expression of TNF-α and IL-6 in dose-dependent manner in MPMs. These data indicated that inhibition of EGFR could prevent LPS-induced inflammation in macrophages, which is NF-κB independent.

### Pharmacological and genetic EGFR inhibition decreased LPS-stimulated inflammatory gene production in BEAS-2B cells

**Figure 2:**
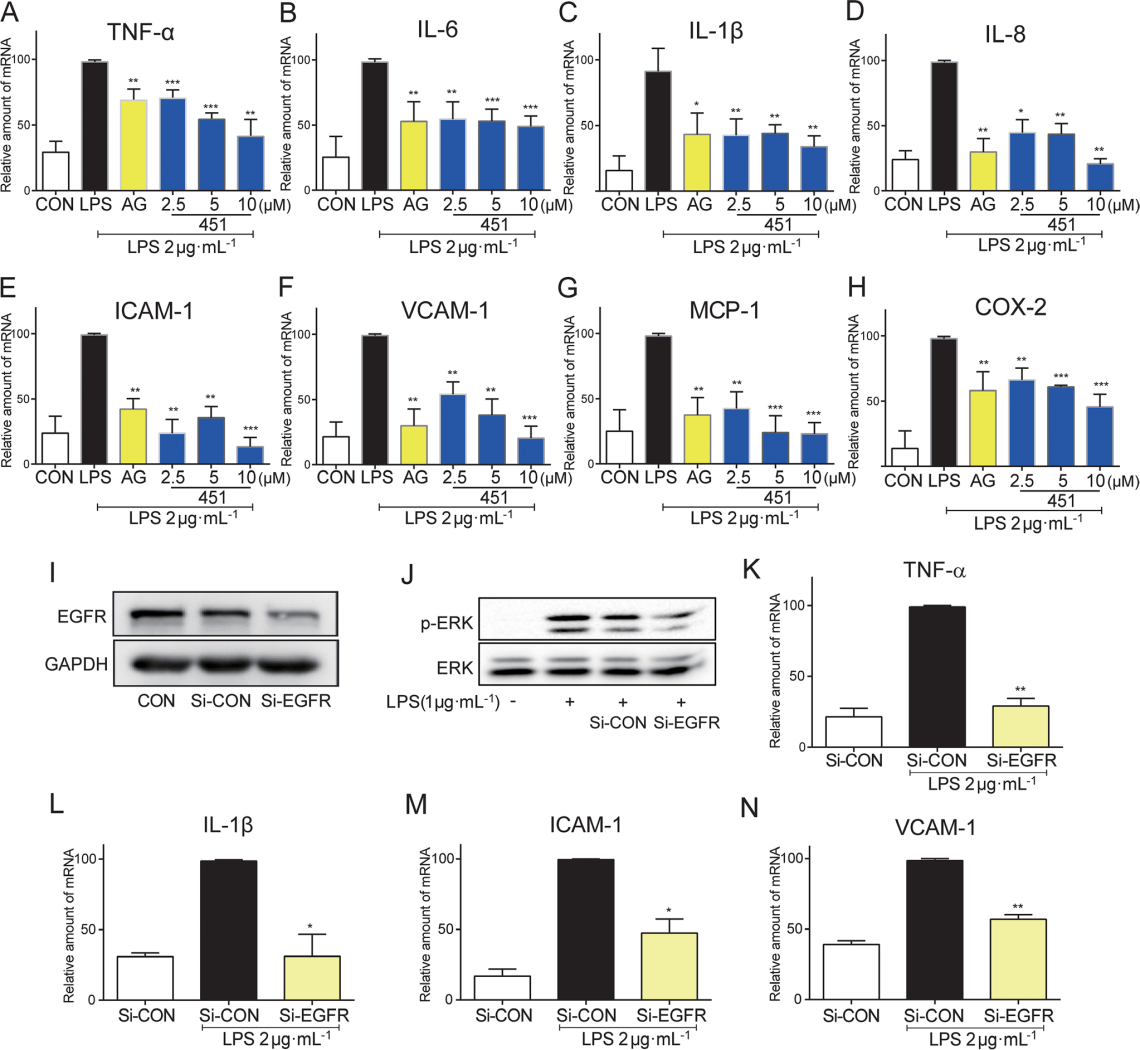
EGFR inhibition reduced the LPS-induced inflammation in BEAS-2B (**A**–**H**) BEAS-2B cells were pre-treated with AG1478 at 10 μM or 451 at various doses (2.5, 5, 10 μM) or vehicle (DMSO) for 30 min prior to stimulation with LPS (2 μg·mL^−1^) for 12 h. Total mRNA was extracted from the cell using TRIzol and the mRNA levels of TNF-α (A), IL-6 (B), IL-1β (C), IL-8 (D), ICAM-1 (E), VCAM-1 (F), MCP-1 (G) and COX-2 (H) were detected by real-time RT-qPCR analysis. (**I**) Western Blot shows EGFR knockdown efficiency following EGFR siRNA (Si-EGFR) transfection in BEAS-2B cells as measured by EGFR protein levels (CON: non transfected cells; Si-CON: non-EGFR scrambled transfection cells). (**J**) Effects of EGFR knock-down by siRNA on ERK phosphorylation in BEAS-2B cells stimulated with 1 μg/mL LPS. (**K**–**N**) Effects of EGFR knock-down by siRNA on inflammatory cytokines TNF-α (K) and IL-1β (L), and adhesion molecular ICAM-1 (M) and VCAM-1 (N) mRNA expression in BEAS-2B cells stimulated with 2 μg·mL^−1^ LPS. Bars represent the mean ± SEM of more than three independent experiments performed in duplicate, and asterisks indicate significant inhibition (*p < 0.05, **p < 0.01, and ***p < 0.001, vs. LPS group).

We further confirmed the anti-inflammatory effect of EGFR inhibitors in human bronchial epithelium BEAS-2B cells. BEAS-2B cells were stimulated with LPS for 12 h after 0.5 h pre-incubation with 451 or AG1478, and the mRNA levels of inflammatory genes were analyzed by real-time qPCR assay. As shown in Figure 2, LPS induced a significant increase in the mRNA expression of pro-inflammatory cytokines, including TNF-α (A), IL-6 (B), IL-1β (C), and IL-8 (D), adhesion molecules ICAM-1 (E) and VCAM-1 (F), chemokine MCP-1 (G), and inducible enzyme COX-2 (H). In contrast, AG1478 at 10 μM and 451 dose-dependently decreased the expression of those transcripts, indicating that EGFR inhibition had also anti-inflammatory effects in lung epithelium cells.

To avoid the nonspecific inhibition of small-molecule inhibitors and confirm the role of EGFR in LPS-induced inflammation, we constructed a genetic silencing of EGFR using siRNA (si-EGFR) in BEAS-2B cells. Compared with scrambled vector, transfection of cells with specific siRNA against EGFR reduced EGFR protein expression by more than 70% (Figure 2I) in BEAS-2B cells and remarkably reduced the phosphorylation of downstream ERK1/2 (Figure 2J). As expected, EGFR silencing significantly blocked LPS-induced mRNA expression of pro-inflammatory cytokines TNF-α (Figure 2K) and IL-1β (Figure 2L), and adhesion molecules ICAM-1 (Figure 2M) and VCAM-1 (Figure 2N) in BEAS-2B cells, validating the role of EGFR in mediating LPS-induced inflammation.

### LPS-induced inflammation in BEAS-2B cells was regulated via EGFR

**Figure 3:**
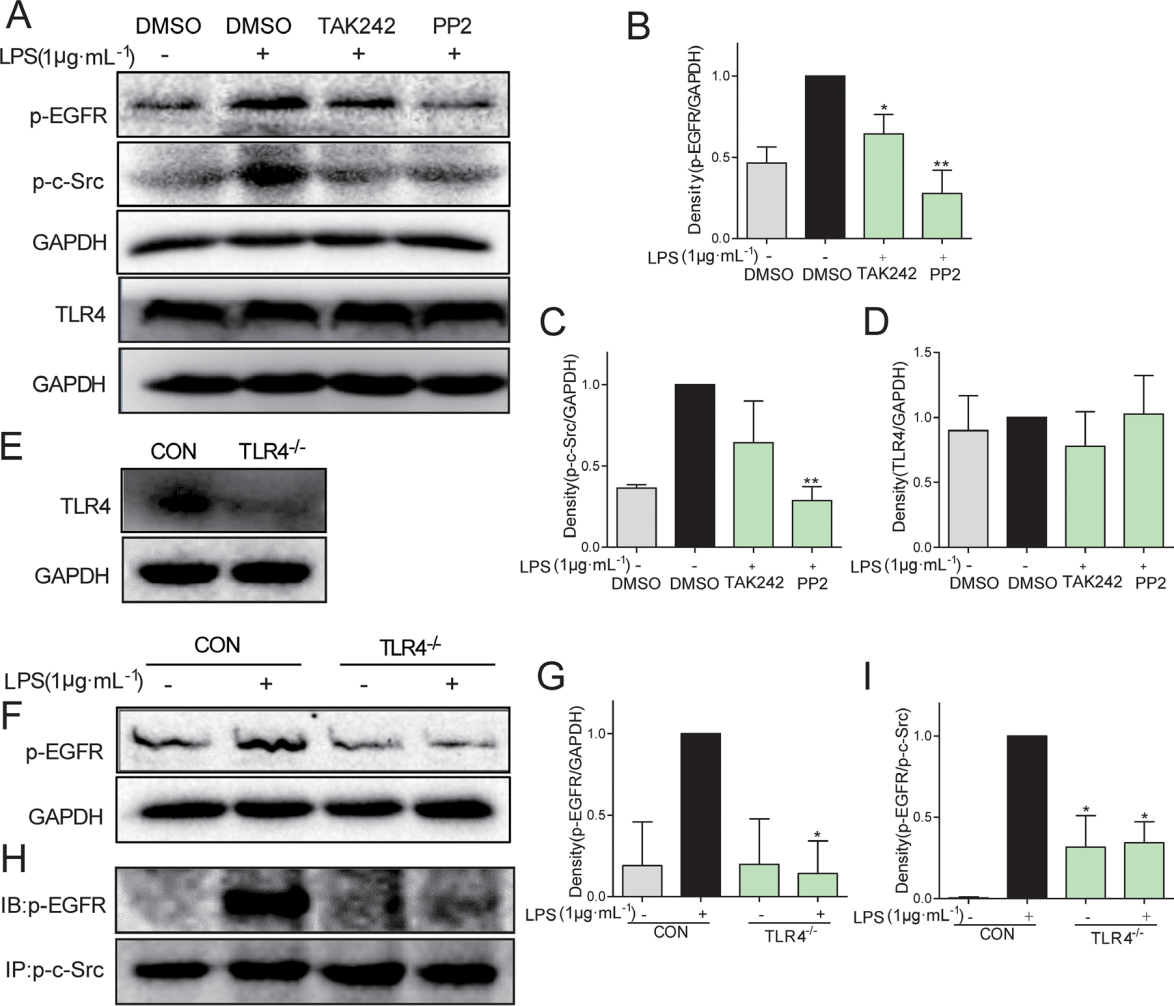
C-Src mediated TLR4-dependent EGFR transactivation in MPMs exposed to LPS (**A**–**D**) MPMs were pre-treated with 10 μM TAK242 or 10 μM PP2 or vehicle (DMSO) for 30 min prior to stimulation with LPS (1 μg·mL^−1^) for 30 min. The total protein was collected and the levels of p-EGFR, p-c-Src, TLR4, and GAPDH were detected by Western Blot assay. (**E**) The TLR4 protein level was detected in MPMs acquired from TLR4^−/−^ mice. (**F** and **G**) MPMs were extracted from WT mice and TLR4^−/−^ mice, respectively. WT MPMs and TLR4^−/−^ MPMs were treated with LPS (1 μg·mL^−1^) for 30 min to detect the EGFR phosphorylation level using Western Blot assay. (**H** and **I**) MPMs were extracted from WT mice and TLR4^−/−^mice, respectively. WT MPMs and TLR4^−/−^ MPMs were treated with LPS (1 μg·mL^−1^) for 5 min to detect the level of p-EGFR/p-c-Src complex using immunoprecipitation assay. Each experiment was detected at least three times. Bars represent the mean ± SEM of more than three independent experiments performed in duplicate, and asterisks indicate significant inhibition (*p < 0.05, **p < 0.01, vs. LPS group).

Further, we investigated whether and how LPS induced EGFR phosphorylation. Toll-like receptor 4 (TLR4) is the classical receptor of LPS in innate immunity. In addition, previous studies suggested that c-Src plays an important role in Ang II-induced EGFR transactivation in type 1 diabetic mice [18]. Two specific small-molecule inhibitors, TAK242 and PP2, were used to block TLR4 and c-Src signaling, respectively. As shown in Figure 3A, pretreatment with either TAK242 or PP2 remarkably inhibited EGFR phosphorylation in LPS-stimulated MPMs, indicating that both TLR4 and c-Src mediated LPS-induced EGFR activation. Additionally, TLR4 inhibition by TAK242 also prevented LPS-induced c-Src phosphorylation, suggesting that the TLR4 was an upstream regulator of c-Src/EGFR signaling (Figure 3A–3D). To validate these results, we isolated the MPMs from TLR4 knockout mice, which showed very low TLR4 expression (Figure 3E). As expected, TLR4^−/−^ MPMs showed no EGFR phosphorylation when exposed to LPS (Figure 3F and 3G). Importantly, immunoprecipitation assay showed a strong interaction between p-c-Src and p-EGFR under LPS stimulation, while TLR4 deletion totally blocked the binding of p-EGFR on p-c-Src (Figure 3H and 3I). These data indicated that c-Src mediated TLR4-dependent EGFR transactivation via directly interaction with EGFR in MPMs exposed to LPS.

### Administration with AG1478 and 451 protected rats from LPS-induced lung injury

**Figure 4:**
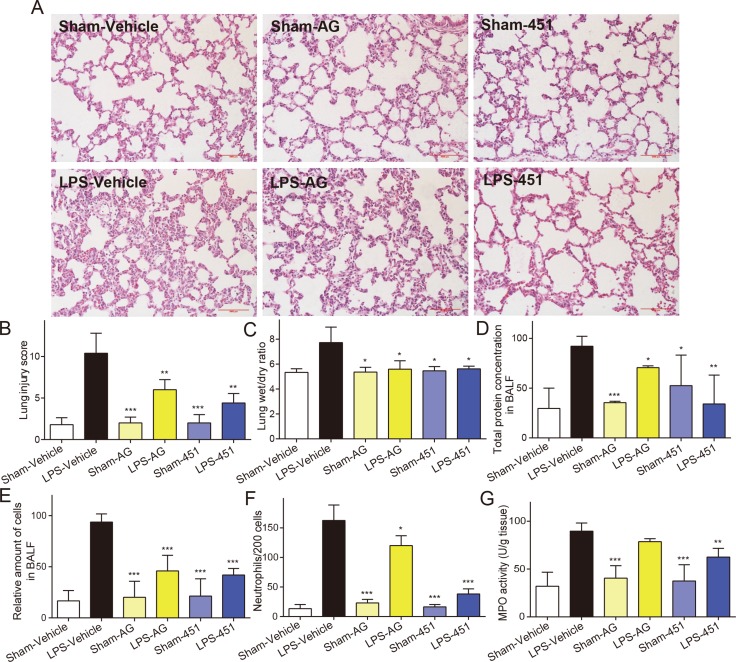
EGFR inhibitors attenuated the LPS-induced acute lung injury in rats SD rats were treated intragastrically with compound AG1478 or 451 at a dosage of 20 mg·kg^−1^ b.wt or vehicle (0.5% CMC-Na) for one week, then challenged with or without 5 mg·kg^−1^ LPS. Rats were euthanized with ketamine after 24 h of LPS induction. (**A**) Histopathological changes in lung tissues determined by H&E staining. (**B**) The lung injury score was determined. (**C**) Lung wet/dry ratio was detected. (**D**–**F**) BAL fluid was collected, and protein content (D), total cells (E) and neutrophils number (F) were measured. (**G**) MPO activity in lung tissue homogenates was measured. Data are mean ± SEM of 4-6 separate animals. *p< 0.05, **p < 0.01 and ***p < 0.001 vs. only-LPS stimulated group.

To understand the effect of EGFR inhibition on LPS-induced ALI, rats were administered with AG1478 or 451 before intratracheal instillation of LPS. Lung tissues were harvested 24 hours after LPS instillation were subjected to H&E staining for histological changes. Figure 4A showed normal pulmonary histology in Sham-vehicle, Sham-AG, and Sham-451 groups, indicating that AG1478 and 451 showed no obvious lung toxicity. Tissues from LPS-vehicle group showed infiltration of inflammatory cells in lung interstitium and alveolar spaces as well as alveolar wall thickening and congestion, while AG1478 or 451 pretreatment markedly ameliorated these pathological changes induced by LPS (Figure 4A). The lung injury score, semiquantitativly assessed by a blinded pathologist, further revealed the protective effects of AG1478 and 451 on LPS-induced ALI (Figure 4B). Figure 4C showed that the increased lung wet/dry weight ratio in the LPS-vehicle group and pretreatment with AG1478 or 451 significantly reduced it. LPS instillation also remarkably increased the total protein concentration in BALF and administration of EGFR inhibitors evidently eliminate the LPS-induced increase in protein concentration (Figure 4D). LPS exposure caused an increase influx of total cells (Figure 4E) and neutrophils (Figure 4F) into BALF, whereas AG1478 or 451 administration reduced the LPS-mediated inflammatory cells infiltration. Furthermore, we measured the ability of EGFR inhibition to influence MPO activity, which is a marker for polymorphonuclear neutrophil (PMN) infiltration and activation. Figure 4G showed that MPO activity was significantly upregulated in the lungs of LPS-challenged rats, while the upregulation of MPO activity was prevented in animals pretreated with AG1478 or 451. These data demonstrated the protective effects of EGFR inhibitors against LPS-induced ALI in rats.

### AG1478 and 451 inhibited EGFR activation in lung tissues of LPS-induced rats

**Figure 5:**
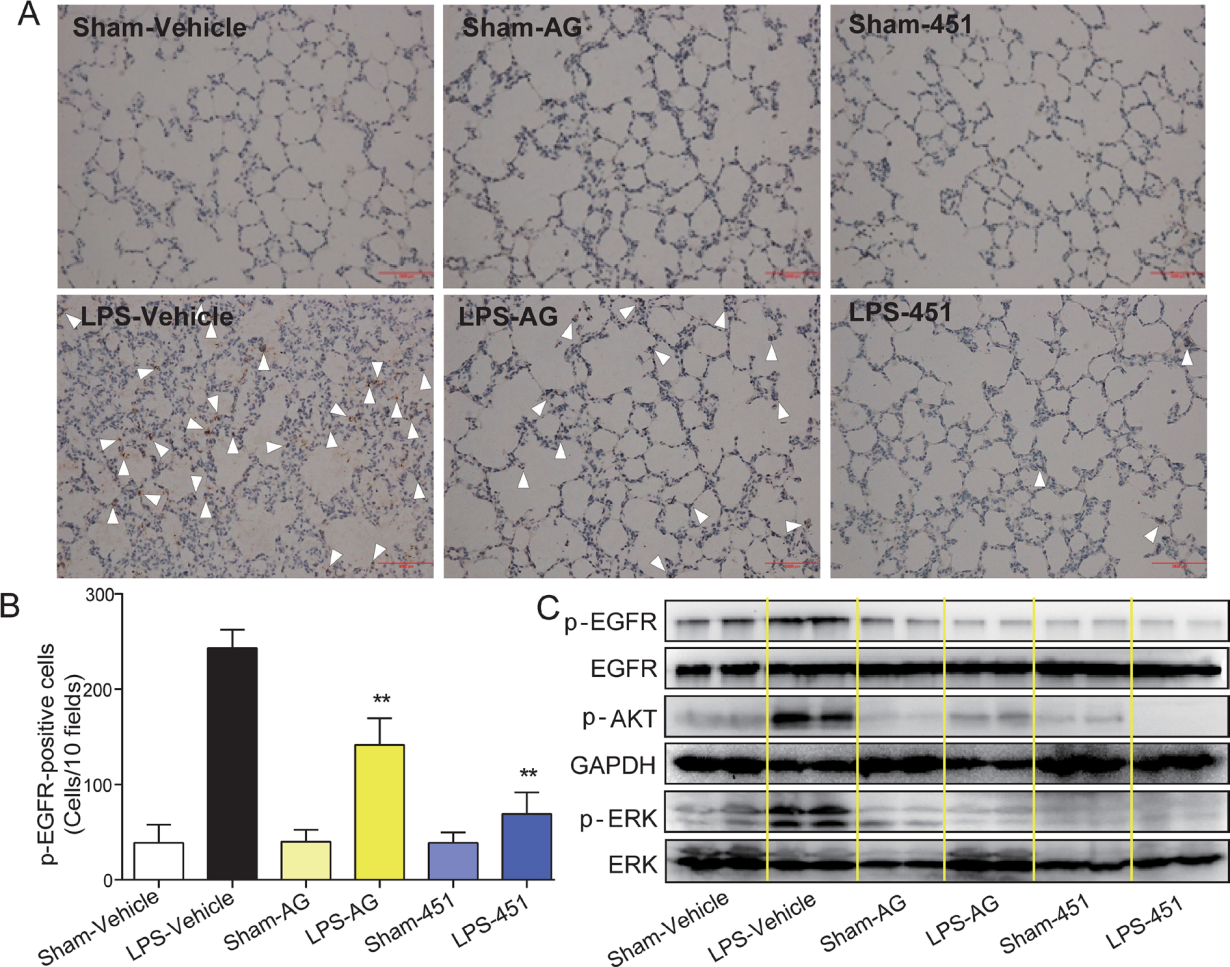
AG1478 and 451 reduced LPS-induced EGFR activation in rats SD rats were treated intragastrically with compound AG1478 or 451 at a dosage of 20 mg·kg^−1^ b.wt or vehicle (0.5% CMC-Na) for one week, then challenged with or without 5 mg·kg^−1^ LPS. Rats were euthanized with ketamine after 24 h of LPS induction. (**A**) EGFR phosphorylation in lung tissue was detected by p-EGFR immunohistochemical assay. (**B**) P-EGFR positive cells were counted in 10 random fields. (**C**) The levels of EGFR signaling pathway in lung tissue was detected by Western Blot assay. Data are mean ± SEM, and **p < 0.01 vs. only-LPS stimulated group.

We examined the level of EGFR phosphorylation in rat lung tissues. The immunohistochemical analysis showed that the p-EGFR level (brown points identified by white arrows) was significantly increased in lung tissues of the LPS-vehicle group. Treatment with AG1478 or 451 remarkably reduced LPS-induced EGFR activation in rat lung (Figure 5A). The levels of p-EGFR in the six groups were counted and shown in Figure 5B, which showed that 451 had a stronger inhibitory effect on EGFR phosphorylation than AG1478. Similar results were observed by Western blot analysis in Figure 5C, which also showed that AG1478 and 451 significantly blocked the activation of ERK and AKT, two downstream proteins in the EGFR signaling pathway, in lung tissues of LPS-challenged rats.

### EGFR inhibitors attenuated LPS-induced inflammatory cytokine release and macrophages infiltration in rat lung tissues

**Figure 6:**
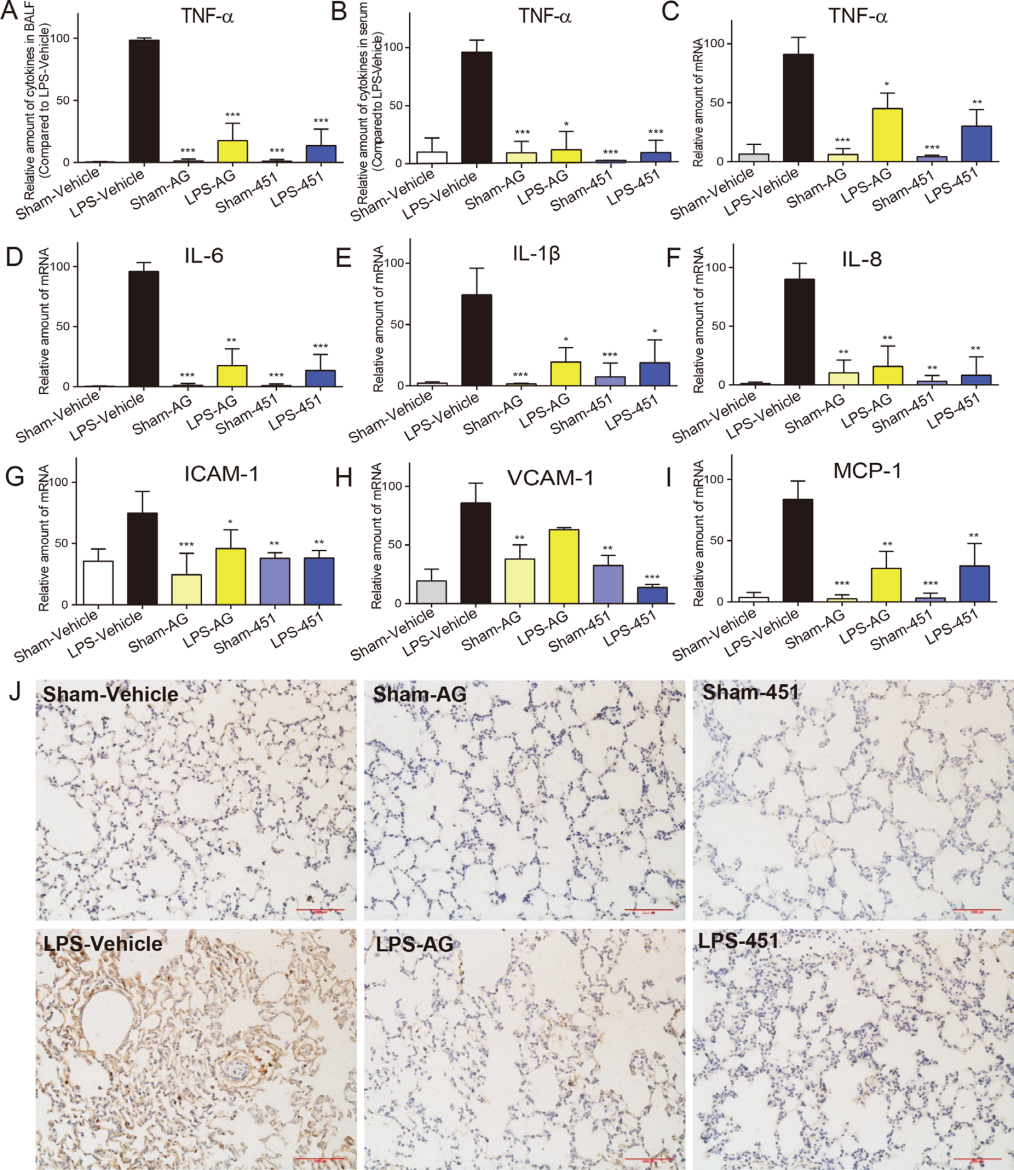
AG1478 and 451 reduced LPS-induced inflammation in rats SD rats were treated intragastrically with compound AG1478 or 451 at a dosage of 20 mg·kg^−1^ b.wt or vehicle (0.5% CMC-Na) for one week, then challenged with or without 5 mg·kg^−1^ LPS. Rats were euthanized with ketamine after 24 h of LPS induction. (**A** and **B**) The inflammatory cytokines TNF-α in BALF (A) and serum (B) were determined by ELISA. (**C**–**I**) Total mRNA was extracted from the lung tissue using TRIzol and the mRNA levels of TNF-α (C), IL-6 (D), IL-1β (E), IL-8 (F), ICAM-1 (G), VCAM-1 (H) and MCP-1 (I) were detected by real-time RT-qPCR analysis. (J) Macrophages infiltration in lung tissue was detected by CD68 immunohistochemical assay. Data are mean ± SEM of 4-6 separate animals. *p < 0.05, **p < 0.01 and ***p < 0.001 vs. only-LPS stimulated group.

Pro-inflammatory cytokines, including TNF-α, IL-1β, IL-6, and IL-8 and cell adhesion molecules, including ICAM-1 and VCAM-1, are major mediators involved in the acute inflammatory response and the recruitment of PMNs into the lungs in LPS-induced pulmonary injury. The TNF-α protein levels in BALF and serum were first determined by ELISA. As shown in Figure 6A and 6B, LPS treatment led to an increase in the concentration of TNF-α in both BALF and serum that was suppressed following the use of EGFR inhibitors. We also tested the mRNA levels of inflammatory cytokines in rat lung tissues by real-time qPCR assay. Figure 6C–6F showed that both EGFR inhibitors decreased the overexpression of TNF-α, IL-1β, IL-6, and IL-8 mRNAs in LPS-challenged rat lung tissues. Similar results were observed in the levels of cell adhesion molecules ICAM-1 (Figure 6G) and VCAM-1 (Figure 6H) and chemokine MCP-1 (Figure 6I). Immunohistochemistry staining for CD68, a marker for macrophage infiltration, was further performed using the lung tissues. As shown in Figure 6J, the ALI rats had a significant increase in CD68-positive macrophages in lung interstitial areas that were markedly attenuated by the treatment with either AG1478 or 451. Generally, 451 showed a stronger anti-inflammatory activity *in vivo* than its lead AG1478. These results indicated that EGFR inhibition reduced the expression of proinflammatory cytokines, which in turn can improve the lung damage caused by LPS-induced ALI.

## DISCUSSION

In the current study, we demonstrate that, for the first time, EGFR inhibitors have protective effects against LPS-induced ALI, validating a critical and detrimental role of EGFR in mediating inflammatory injuries in lung. The major findings of this work demonstrate that EGFR is transactivated by TLR4/c-Src signaling under LPS stimulation. As a result, the downstream AKT and ERK signaling pathways are activated, which contributes to the up-regulation of the expression of a series of inflammatory cytokines and adhesion molecules, and subsequent acute inflammatory injuries in lungs. Accordingly, inhibition of EGFR both *in vitro* and *in vivo* results in decreased inflammation and attenuated lung injuries.

EGFR and its ligands are frequently up-regulated in human cancers [19]. EGFR is also endogenously expressed in numerous cell types and is an important factor in the control of many fundamental cellular processes, including the cell migration, cell cycle, cell metabolism and survival, in addition to cell proliferation and differentiation [19]. Over the past decade, research into the EGFR pathway has revealed its roles in inflammation and oxidative stress. Furthermore, EGFR inhibition by pharmacological or genetic methods was found to attenuate chronic diseases, such as insulin resistance, diabetic complications, atherosclerosis, and cardiovascular diseases, which generally accompanied with the attenuation of chronic inflammation [20–22].

TLR activation initiates a complex and integrated signaling cascade that activates EGFR in airway epithelial cells [23]. For instance, TLR3 activation requires EGFR tyrosine kinase [24]. Increasing evidences demonstrate that EGFR regulates LPS-induced and TLR4-mediated inflammatory response. Lu et al. found that LPS stimulated EGFR activation in macrophages, which regulated cytokine production and experimental colitis [25]. Deletion of EGFR in LPS-stimulated macrophages leads to increases in anti-inflammatory cytokine IL-10, which plays a role in suppressing pro-inflammatory cytokine production, resulting in protection of mice from intestinal inflammation [26]. EGFR signaling also mediates LPS-induced adhesion molecule expression and neutrophil adhesion, as evidenced in human tracheal smooth muscle cells [27]. Recently, the ability of TLR4 to activate NF-κB in response to LPS has been found to be impaired by down regulating EGFR expression or by using the EGFR inhibitor erlotinib [28]. Almost simultaneously, another independent group confirmed this by that administration of an EGFR inhibitor to mice protects them from LPS-induced septic shock and death. Nevertheless, they found that EGFR inhibition only inhibited LPS-induced IFN-regulatory factor (IRF)-driven gene expression through the PI3 kinase/AKT pathway, but not NF-κB-mediated TNF-α/IL-6 overproduction in myeloid cells and mice [29]. Our results in this study continue to provide evidence for the involvement of EGFR signaling in LPS-induced inflammation in MPMs, BEAS-2B cells, and rats. However, our data showed EGFR inhibition reduced TNF-α/IL-6 overproduction, but did not affect NF-κB activation. The reduction of inflammatory cytokines by EGFR inhibition seems to be associated with the inhibition of ERK/AKT signaling activation (Figures 1 and 5), which are partly consistent with both previous publications [28, 29]. Thus, the mechanism by which EGFR mediates LPS-induced cytokine expression seems to be complicated and requires further demonstration in different experimental cell types.

It has been demonstrated that Src kinase family members mediates EGFR activation [30, 31]. A Src family member is known to be involved in LPS-dependent NF-κB activation [32]. Further, LYN kinase, a Src family member, is also required in LPS/TLR4-EGFR signaling cascade in human epithelial cells [28]. We also examined whether EGFR activation was dependent on Src. Consistently, both TAK242 and PP2 effectively inhibited EGFR and c-Src phosphorylation (Figure 3) and TLR4 knockout abolished EGFR activation and the interaction of p-EGFR and p-c-Src. These results validated that TLR4 activated EGFR via c-Src signaling in human lung epithelial BEAS-2B cells.

Although some ventilator therapies have improved outcomes for ALI, the majority of therapies for ALI/ARDS have largely been unsuccessful [33]. Pharmacologic therapies, such as corticosteroids, surfactants, and nitric oxide have also been proven ineffectual in treatment of ALI [34]. ALI and ARDS result from an acute systemic inflammatory process that results in pulmonary infiltrate, edema, and hypoxia [35]. This process is characterized at the cellular level by loss in integrity of the alveolar-capillary membrane, excessive neutrophil infiltration, and release of pro-inflammatory chemokines (chemotaxic cytokines) [35]. The increase in proinflammatory cytokines serves as both a response and a marker for the ongoing cellular damage from ALI [2].

Although evidences demonstrated that the use of small-molecule EGFR inhibitors could contribute to the development of a novel strategy for therapeutic intervention to ameliorate septic shock [28, 29], the pharmacological effects of EGFR inhibitors on ALI are still unknown. Moreover, Chika Harada's group found that EGFR inhibitor gefitinib worsened acute lung injury with repairing airway epithelium in mice received intraperitoneally naphthalene [36]. Thus, the beneficial effects of EGFR inhibitors against LPS-induced ALI need to be investigated. Consistent with the anti-inflammatory results in both primary macrophages and bronchial epithelium BEAS-2B cells, the *in vivo* data showed that EGFR inhibitors AG1478 and 451 significantly improved LPS-induced lung inflammation and injuries in rats with intratracheal instillation of LPS. Administration with AG1478 or 451 alone showed no obvious toxicity in control sham rats. These improvements include decreasing infiltration of inflammatory cells in the lung interstitial and alveolar spaces and reducing LPS-induced thickening of the alveolar wall, which resulted in significantly lower lung injury scores. It is noteworthy that EGFR inhibitors, well known drugs used in cancer treatment, may be also beneficial in curing ALI triggered by infection and LPS.

In summary, our results showed that pharmacological inhibition and siRNA silencing of EGFR reduced the LPS-induced inflammatory response *in vitro*. Mechanistically, TLR4 and c-Src mediated LPS-induced activation of EGFR. *In vivo*, oral administration with EGFR inhibitors significantly attenuated the LPS-induced ALI. Thus, TLR4/c-Src-dependent EGFR signaling activation is involved in LPS-induced inflammation and ALI. EGFR inhibition may provide a therapeutic strategy for the treatment of patients suffering from ALI induced by microbial infection. Further investigations with experimental animals will be required for optimizing the efficacy of such therapeutic interventions using clinically used EGFR inhibitors or antibodies.

## MATERIALS AND METHODS

### Reagents

LPS, TLR4 inhibitor TAK242, c-Src inhibitor PP2, AG1478 (abbreviated AG) were purchased from Sigma Chemical Co. (St. Louis, MO). EGFR small interfering RNA (Si-EGFR), Anti-p-EGFR and anti-EGFR were purchased from Cell Signaling (Danvers, MA). Anti-CD68, anti-p-AKT, anti-AKT, Anti-p-ERK, anti-ERK, anti-IκBα, anti-GAPDH, anti-p-c-Src and anti-TLR4 antibodies were obtained from Santa Cruz Biotechnology (Santa Cruz, CA, USA). EGFR inhibitor 451, provided by our own lab with HPLC purity > 99%, was identified using ESI-MS and dissolved in dimethyl sulfoxide (DMSO) for *in vitro* experiments and in 0.5% CMCNa for *in vivo* experiments.

### Cell culture

Human lung epithelial cells BEAS-2B were purchased from Shanghai Institute of Biosciences and Cell Resources Center (Chinese Academy of Sciences, Shanghai, China). BEAS-2B cells were cultured in a 5% CO_2_ atmosphere with 100 U·mL^−1^ penicillin and 100 U·mL^−1^ streptomycin. BEAS-2B cells were cultured in RPMI-1640 medium (Gibco, Eggenstein, Germany) with the 20% FBS (Gibco, Eggenstein, Germany). Mouse peritoneal macrophages (MPMs) from ICR mice were obtained and prepared as described in our previous paper [37].

### Animals

Male ICR mice (*n* = 23) weighing 18–22 g and Sprague-Dawley (SD) rats (*n* = 48) weighing 180–220 g were obtained from the Animal Center at Wenzhou Medical University (Wenzhou, China). All animals were housed at a constant room temperature with a 12:12 h light-dark cycle and fed with a standard rodent diet and water. All animal care and experimental procedures complied with the ‘The Detailed Rules and Regulations of Medical Animal Experiments Administration and Implementation’ (Order No. 1998-55, Ministry of Public Health, China) and ‘Ordinance in Experimental Animal Management’ (Order No. 1998-02, Ministry of Science and Technology, China) and were approved by the Wenzhou Medical University Animal Policy and Welfare Committee (Approval Document No.wydw2014-0062).

### LPS-induced ALI/ARDS in rats

AG1478 and 451 were firstly dissolved in 0.5% CMC-Na, which served as vehicle 1. SD rats weighing 180-220 g were pretreated with AG1478 and 451 (20 mg·kg^−1^, 0.5% CMC-Na) for 7 consecutive days by intragastric administration before intratracheal instillation of LPS (5 mg·kg^−1^, 0.9% saline). Simultaneously, control animals (sham rats) received a similar volume of treatment with either vehicle 1 (0.5% CMC-Na), AG1478 or 451 before intratracheal instillation of vehicle 2 (0.9% saline solution). Thus, the SD rats were randomly divided into six groups (*n* = 8 per group): 3 sham rat groups: treated with vehicle 1 and 2 (Sham-Vehicle group), treated with AG1478 (Sham-AG group), and treated with 451 (Sham-451 group) and three LPS-induced ALI/ARDS groups: pretreated with vehicle 1 (LPS-Vehicle group), pretreated with AG1478 (LPS-AG group), pretreated with 451 (LPS-451 group).

For LPS-induced ALI/ARDS evaluation, rats were sacrificed under ether anesthesia 24 h after LPS (5 mg·kg^−1^) instillation. Blood samples were then collected from the aorta ventral using a heparinized syringe with a needle and centrifuged at 3000 rpm at 4°C for 10 min. The serum was collected for subsequent analysis. To evaluate the severity of ALI/ARDS, the lung wet/dry weight ratio was calculated. The wet lung weight was obtained immediately after dissection, and the dried lung weight was measured after samples were dried to stable weight in the oven at 60°C. The left middle lobe of the right lung was for histological evaluation and immunohistochemistry study, and the lower lobe was frozen in liquid nitrogen for Western blot or PCR analysis. While the right lungs were subjected to bronchoalveolar lavage (BAL).

### BAL analysis

BAL was performed using a tracheal cannula and 3 mL of 0.9% saline injected 3 times. The BAL fluid (BALF) was then centrifuged immediately at 1000 rpm for 5 min at 4°C. The supernatant of BALF was immediately stored at −80°C before examination of cytokine levels and protein concentration for lung permeability. The sediment was resuspended in 50 μL of 0.9% saline for the determination of the total number of cells and neutrophils. The total number of cells in BALF was counted by a standard hemocytometer, and neutrophil cells were confirmed by counting 200 cells on a smear prepared by Wright-Giemsa staining.

### Determination of cytokine level

For the determination of cytokine levels in the cell medium, BALF and rat plasma, cytokine-specific ELISA kits (Bioscience, San Diego, CA) were used according to the manufacturer's instructions. The total amount of TNF-α and IL-6 in the cell medium was normalized to the total protein amount from the viable cell pellets.

### Total protein assay

Total protein content level in BALF was measured by using a Bradford assay (Bio-Rad, Tokyo, Japan) according to the manufacturer's instructions.

### Measurement of myeloperoxidase (MPO) activity

To quantify neutrophil infiltration, MPO activity in the homogenized lung tissues was determined using a MPO Detection Kit (Nanjing Jiancheng Bioengineering Institute, China) as previously described [38]. Briefly, lung tissue was homogenized in 1 mL of 50 mM potassium phosphate buffered saline (PBS, pH 6.0) containing 0.5% hexadecyltrimethylammonium hydroxide and centrifuged at 12,000 rpm at 4°C for 20 min. 10 μL of the supernatant was transferred into PBS (pH 6.0) containing 0.17 mg·mL^−1^ 3,3′-dimethoxybenzidine and 0.0005% H_2_O_2_. MPO activity of the supernatant was determined by using absorbance values measured via spectrophotometry at 460 nm and presented as units per gram of total protein (U·g^−1^). Total protein content in the samples was analyzed using total protein assay.

### Western blot analysis

Either collected cells or homogenized lung tissue samples (80 μg) were lysed, subjected to 10% sodium dodecyl sulfate-polyacrylamide gel electrophoresis, and transferred onto a nitrocellulose membrane (Bio-Rad Laboratory, Hercules, CA). After pre-incubation in blocking buffer (5% milk in tris-buffered saline containing 0.05% Tween 20) for 1.5 h at room temperature, membranes with diverse bands were incubated with specific primary antibodies overnight at 4°C The membranes were then washed with TBST and incubated with secondary horseradish peroxidase-conjugated antibody (Santa Cruz, CA; 1:2000) for 1 h at room temperature. After washing with TBST three times, the membranes were visualized with enhanced chemiluminescence reagents (Bio-Rad, Hercules, CA). The magnitude of the immunoreactive bands was analyzed using Image J software (NIH, Bethesda, MD).

### RNA extraction and real-time quantitative PCR assay

Total RNA was isolated from cells or tissues (50–100 mg) using TRIZOL (Invitrogen, Carlsbad, CA). Reverse transcription and quantitative PCR (RT-qPCR) were performed using M-MLV Platinum RT-qPCR Kit (Invitrogen, Carlsbad, CA). Real-time quantitative PCR was carried out using the Eppendorf Realplex 4 instrument (Eppendorf, Hamburg, Germany). Primers of genes including TNF-α, IL-6, IL-1β, IL-8, ICAM-1, VCAM-1, MCP-1, COX-2, and β-actin were obtained from Invitrogen (Invitrogen, Shanghai, China). The primer sequences used were shown in Table 1. The relative amount of each gene was normalized to the amount of β-actin.

**Table 1:** Primer sequences for real-time quantitative PCR

Gene	Species	FW	RW
TNF-α	human	CCCAGGGACCTCTCTCTAATC	ATGGGCTACAGGCTTGTCACT
IL-6	human	GCACTGGCAGAAAACAACCT	TCAAACTCCAAAAGACCAGTGA
IL-1β	human	ACGCTCCGGGACTCACAGCA	TGAGGCCCAAGGCCACAGGT
IL-8	human	CTCTTGGCAGCCTTCCTGATT	ACTCTCAATCACTCTCAGTTCT
ICAM-1	human	GAACCAGAGCCAGGAGACAC	TCCCTTTTTGGGCCTGTTGT
VCAM-1	human	GGCGCCTATACCATCCGAAA	TATGACCCCTTCATGTTGGC
MCP-1	human	TTCCCCTAGCTTTCCCCAGA	TCCCAGGGGTAGAACTGTGG
COX-2	human	TTCTCCTTGAAAGGACTTATGGGTAA	AGAACTTGCATTGATGGTGACTGTTT
β-Actin	human	CCTGGCACCCAGCACAAT	GCCGATCCACACGGAGTACT
TNF-α	rat	TACTCCCAGGTTCTCTTCAAGG	GGAGGCTGACTTTCTCCTGGTA
IL-6	rat	GAGTTGTGCAATGGCAATTC	ACTCCAGAAGACCAGAGCAG
IL-1β	rat	GGGCCTCAAGGGGAAGAATC	ATGTCCCGACCATTGCTGTT
IL-8	rat	GAAGATAGATTGCACCGATG	CATAGCCTCTCACACACATTTC
ICAM-1	rat	GCCTGGGGTTGGAGACTAAC	CTGTCTTCCCCAATGTCGCT
VCAM-1	rat	AGGTTGGGGATTCCGTTGTT	ACACATTAGGGACCGTGCAG
MCP-1	rat	GTCACCAAGCTCAAGAGAGAGA	GAGTGGATGCATTAGCTTCAGA
β-Actin	rat	AAGTCCCTCACCCTCCCAAAAG	AAGCAATGCTGTCACCTTCCC

### Transient transfection with siRNAs

Human EGFR-specific siRNA and the control siRNA were obtained from GenePharma (Shanghai GenePharma CO., Ltd). Transient transfection of siRNAs was performed using transfection reagent Lipo2000 (Invitrogen, Carlsbad, CA). Briefly, BEAS-2B cells were subjected to the mixture of siRNA and Lipo2000 pre-formulated in RPMI-1640 medium without FBS and antibiotics. After incubated for 6 hours, the mixture was removed and the cells were switched to a RPMI-1640 containing 20% FBS and 1% antibiotics. Another 18 h continuing culture was needed before cells were treated with LPS for the following Western blot or ELISA analysis.

### Lung histopathology

Lung tissues, fixed in 4% paraformaldehyde and embedded in paraffin, were cut into 5 μm thick sections and subsequently stained with hematoxylin and eosin (H&E) stain for histological analysis. A pathologist blindly scored each lung injury according to the following four categories: alveolar congestion, hemorrhage, neutrophil infiltration into the airspace or vessel wall, and thickness of alveolar wall/hyaline membrane formation. Each category was graded on a 0- to 4-point scale: 0 = no injury; 1 = injury up to 25% of the field; 2 = injury up to 50% of the field; 3 = injury up to 75% of the field; and 4 = diffuse injury [39]. The lung-injury score for each animal was calculated as the mean of four lung sections under light microscopy (200× amplification; Nikon).

### Immunohistochemistry

Tissue sections (5 μm thickness) were prepared, deparaffinized in xylene, and hydrated using an ethanol gradient. A Pressure-cooker was used for heat- induced antigen retrieval (10 mM sodium citrate buffer, pH 6.5). After treatment with 30% of hydrogen peroxide, all sections were blocked in 5% bovine serum albumin (BSA) and incubated with primary anti-CD68 antibody overnight at 4°C. The slides were then incubated with HRP-labeled secondary antibody for 10 min. After the sections were incubated with 3,3-diaminobenzidine tetrahydrochloride (DAB) for color development and counterstained with hematoxylin, the slides were evaluated under a microscope (200× amplification; Nikon). The percentage of CD68-positive inflammatory cells was calculated in 10 randomly chosen fields (200×) per section.

### Statistical analysis

Data was presented as mean ± SEM. The statistical significance between groups was obtained by Student's *t-test* or ANOVA multiple comparisons in GraphPad Pro 6.0 (GraphPad, San Diego, CA, USA). *P* < 0.05 was considered to be of statistical significance and denoted as *. All the experiments related to cells were repeated at least three times. *In vivo* experiments were performed with *n* ≥ 5 rats in each group.

## Abbreviations

451: N-(4-((1H-indol-5-yl)amino)quinazolin-6-yl)acrylamide
AG1478: N-(3-chlorophenyl)-6,7-dimethoxyquinazolin-4-amine
AKT: protein kinase B
ALI: acute lung injury
ARDS: acute respiratory distress syndrome
BALF: bronchoalveolar lavage fluid
BSA: bovine serum albumin
CMCNa: sodium carboxymethyl cellulose
COX-2: cyclooxygenase-2
DAB: 3,3-diaminobenzidine tetrahydrochloride
DMSO: dimethyl sulfoxide
EGFR: epidermal growth factor receptor
ELISA: enzyme-linked immunosorbent assay
ERK: extracellular regulated kinase
FBS: fetal calf serum
GAPDH: glyceraldehyde-3-phosphate dehydrogenase
H&E: hematoxylin and eosin
H_2_O_2_: hydrogen peroxide
HPLC: high performance liquid chromatography
ICAM-1: intercellular adhesion molecule-1
ICR: institute of cancer research
IL-6: interleukin-6
IRF: IFN-regulatory factor
IκB: inhibitor κ-gene binding
LPS: lipopolysaccharides
MAPK: mitogen-activated protein kinase
MCP-1: monocyte chemoattractant protein-1
MPMs: mouse peritoneal macrophages
MPO: myeloperoxidase
MV: mechanical ventilation
NF-κB: nuclear factor-κ-gene binding
PBS: phosphate buffered saline
PI3K: phosphoinositide-3 kinase
PMN: polymorphonuclear neutrophil
PP2: 1-(tert-butyl)-3-(4-chlorophenyl)-1H-pyrazolo[3,4-d]pyrimidin-4-amine
Ras: rat sarcoma
RT-qPCR: reverse transcription and quantitative polymerase chain reaction
SD: Sprague-Dawley
SEM: standard error of mean
sh-RNA: short hairpin ribonucleic acid
Src: sarcomagene
TAK242: ethyl (S)-6-(N-(2-chloro-4-fluorophenyl)sulfamoyl)cyclohex-1-ene-1-carboxylate
TBST: tris-buffered saline tween
TLR4: toll-like receptor 4
TNF-α: tumor necrosis factor-α
VCAM-1: vascular cell adhesion molecule-1
